# Genomic Ancestry, Self-Rated Health and Its Association with Mortality in an Admixed Population: 10 Year Follow-Up of the Bambui-Epigen (Brazil) Cohort Study of Ageing

**DOI:** 10.1371/journal.pone.0144456

**Published:** 2015-12-17

**Authors:** M. Fernanda Lima-Costa, James Macinko, Juliana Vaz de Melo Mambrini, Cibele C. Cesar, Sérgio V. Peixoto, Wagner C. S. Magalhães, Bernardo L. Horta, Mauricio Barreto, Erico Castro-Costa, Josélia O. A. Firmo, Fernando A. Proietti, Thiago Peixoto Leal, Maira R. Rodrigues, Alexandre Pereira, Eduardo Tarazona-Santos

**Affiliations:** 1 Fundação Oswaldo Cruz, Instituto de Pesquisas Rene Rachou, Belo Horizonte, Minas Gerais, Brazil; 2 University of California, Los Angeles, Fielding School of Public Health, Departments of Health Policy and Management and Community Health Sciences, Los Angeles, California, United States; 3 Universidade Federal de Minas Gerais, Departamento de Estatística, Belo Horizonte, Minas Gerais, Brazil; 4 Universidade Federal de Minas Gerais, Departamento de Biologia Geral, Belo Horizonte, Minas Gerais, Brazil; 5 Universidade Federal de Pelotas, Departamento de Medicina Social, Pelotas, Rio Grande do Sul, Brasil; 6 Fundação Oswaldo Cruz, Instituto de Pesquisas Gonçalo Muniz, Bahia, Salvador, Brasil; 7 Universidade de São Paulo, Instituto do Coração, São Paulo, Brazil; University of Bristol, UNITED KINGDOM

## Abstract

**Background:**

Self-rated health (SRH) has strong predictive value for mortality in different contexts and cultures, but there is inconsistent evidence on ethnoracial disparities in SRH in Latin America, possibly due to the complexity surrounding ethnoracial self-classification.

**Materials/Methods:**

We used 370,539 Single Nucleotide Polymorphisms (SNPs) to examine the association between individual genomic proportions of African, European and Native American ancestry, and ethnoracial self-classification, with baseline and 10-year SRH trajectories in 1,311 community dwelling older Brazilians. We also examined whether genomic ancestry and ethnoracial self-classification affect the predictive value of SRH for subsequent mortality.

**Results:**

European ancestry predominated among participants, followed by African and Native American (median = 84.0%, 9.6% and 5.3%, respectively); the prevalence of Non-White (Mixed and Black) was 39.8%. Persons at higher levels of African and Native American genomic ancestry, and those self-identified as Non-White, were more likely to report poor health than other groups, even after controlling for socioeconomic conditions and an array of self-reported and objective physical health measures. Increased risks for mortality associated with worse SRH trajectories were strong and remarkably similar (hazard ratio ~3) across all genomic ancestry and ethno-racial groups.

**Conclusions:**

Our results demonstrated for the first time that higher levels of African and Native American genomic ancestry—and the inverse for European ancestry—were strongly correlated with worse SRH in a Latin American admixed population. Both genomic ancestry and ethnoracial self-classification did not modify the strong association between baseline SRH or SRH trajectory, and subsequent mortality.

## Introduction

Self-rated health (SRH) is one of the most widely used epidemiologic variables because of its simplicity, its validity in different contexts and cultures, and its strong predictive power for future mortality. [1–4] While there is considerable evidence of ethnoracial disparities in SRH in the USA [5–7], studies conducted in Latin American countries have reported inconsistent results. [8–10] One possible explanation for these inconsistencies is the complexity surrounding ethnoracial classification in Latin American populations. [11]

Brazil, the world’s fifth most populous nation [12], offers a unique opportunity to explore the extent of agreement among objective measures of ethnoracial background (genome-wide ancestry) and ethnoracial identity on SRH disparities. The Brazilian population originated from African, European and Native American ancestral roots. [11, 13] The slave trade to Brazil was the largest in the Americas; 3.6 million African slaves were brought to Brazil, seven times more than to the United States. [11] The absence of legal segregation and other factors resulted in the construction of a complex, fluid system of ethnoracial classification. [11, 13] A recent population-based multicenter study concluded that ethnoracial self-classification in Brazil is affected by both genomic ancestry and non-biological factors [13]

To our knowledge, no previous study has examined the association between genome-wide ancestry and SRH in an admixed Western population. We used 370,539 SNPs to examine the association between individual genomic proportions of African, European and Native American ancestry, as well as that of ethnoracial self-classification, with SRH in a population-based cohort of older adults in Brazil. Additionally, we examined whether genomic ancestry and ethnoracial self-classification affect the predictive value of SRH for subsequent mortality.

## Materials and Methods

### Ethics Statement

The Bambui cohort study of aging was fully approved by the Comissão de Ética em Pesquisa (Institutional Review Board) at Oswaldo Cruz Foundation, Rio de Janeiro, Brazil. Written informed consent was obtained from all participants at baseline and at all follow-up interviews. Genotyping was approved by Brazil’s national research ethics committee, as part of the Epigen-Brazil protocol (CONEP, resolution 15895).

### Study design and population

The Bambui cohort study of aging is ongoing in Bambuí, a city of approximately 15,000 inhabitants in the state of Minas Gerais in Southeast Brazil. [14] The population eligible for the cohort consisted of all residents aged 60 years and over on 1 January 1997 (1,606 out of 1,742 of whom participated). Cohort members undergo annual follow-up visits, which consist of an interview and verification of death certificates (98.8% of which have been verified). All deaths from any cause occurring from study enrollment to December 31, 2007, were included in this analysis. At baseline, 1,442 participants had their DNA stored and authorized its use for future investigation. Genotyping were performed in 2012, as part of the Epigen-Brazil Initiative. [13]

### Self-rated health

Information on SRH was obtained during the household interview at baseline and at each subsequent wave. SRH was measured by asking the question, ‘‘In general, would you say your health is …,” and five response categories ranged from ‘‘excellent,” to “very poor”. Language plays a role in an individual´s choice on the SRH scale [15, 16] and we have shown that for the Bambui cohort population, whose language is Portuguese, the middle option (“fair”) seems to describe normal health and is viewed as similar to the “good” option. [4, 17] Thus, we categorized SRH as a binary variable, comprising poor/very poor (exposure category) versus fair/good/excellent.

### Genotyping and external parental populations

Participants were genotyped by the Illumina facility, using the Omni 2.5M array (Illumina, San Diego, California). We performed the unsupervised tri-hybrid (k = 3) admixture analyses based on 370,539 SNPs shared between samples from the Epigen-Brazil study population, the HapMap Project, and the Human Genome Diversity Project (HGDP). [18, 19] As external panels, we used the following HapMap samples: 266 Africans, 262 Europeans (American and Italian), 77 admixed Mexican Americans, 83 African Americans, and 93 Native Americans from the HGDP. Further details can be seen elsewhere. [13]

### Ethnoracial self-classification

The Brazilian census uses ethnoracial self-classification with five groups: White, Brown/Mixed (“pardo” in official Portuguese), Black, “Yellow” (Asian) and Indigenous (Native American). [12] At baseline, cohort participants categorized themselves into the above mentioned ethnoracial groups, according to standard photographs of Brazilians; no individuals categorized themselves as Native American or Asian. Those self-classified as Black or Brown/Mixed were considered as Non-White in the current analysis.

### Other baseline measures

SRH is affected by several main factors [2] and variables selected for this study followed this framework, including: 1) socio demographic characteristics (age, sex, schooling and household income); 2) lifestyle (current smoking and leisure-time physical activity); 3) mental symptoms (common mental disorders); 4) physical functioning (disability in activities of daily living); and 5) objective measures of health (described below).

Based on its distribution, we categorized schooling into incomplete primary school (<4 years) and complete primary and higher (4 years and more). We categorized monthly household income per capita into equal or superior to the median value (median = 1.5 Brazilian minimum wage or USD 180.00 at baseline) and lower. Current smokers were persons who had smoked at least 100 cigarettes during their lifetime and who were still smokers. Leisure-time physical activity was defined as activity of any intensity for 20–30 minutes at least 3 times a week during the previous 3 months. Assessment of common mental disorders was based on participants’ answers to the 12-item version of the General Health Questionnaire. A score of 4/5 was used to define exposure status, as recommended for the study population. [20] Participants were considered to have a disability in activities of daily living if they reported much difficulty or inability in at least one of the following: feeding oneself, dressing oneself, bathing or showering, using the toilet, getting in and out of the bed to a chair, and/or walking across a room. [17]

Objective measures of health were those previously found to be associated with increased risk of mortality among cohort participants. [21–25] Systolic blood pressure was defined as the mean of 2 out of 3 measures according to standard protocols. Body mass index was defined as weight in kilograms divided by the square of height in meters. Blood fasting glucose, total cholesterol, and lipoprotein cholesterol were determined by using standard enzymatic methods (Merck, Darmstadt, Germany). Plasma Brain Natriuretic Peptide (BNP) was measured using a micro particle-based immunoassay (AxSYM MEIA; Abbott Laboratories, Inc., Abbott Park, Illinois). White blood cell count and hemoglobin level were assessed using an electronic counter (Coulter Counter T 890; Coulter Electronics, Hialeah, Florida). Infection with *Trypanosoma cruzi* was assessed by means of 1 hemagglutination assay (Biolab Mérieux SA, Rio de Janeiro, Brazil) and 2 enzyme-linked immunosorbent assays (Abbott Laboratories, Inc., and Wiener Laboratories, Rosario, Argentina) performed concurrently. Blood samples were collected after a 12-hour fast. Further details are described elsewhere. [14]

### Statistical analysis

To estimate the genetic ancestry of the study population, we applied the methodology implemented in the software Admixture [26] and used an unsupervised mode to identify clusters corresponding to the 3 ancestral populations (African, European and Native American) from the genetic structure of our dataset. We estimated kinship coefficients for each possible pair of individuals, using the Related Estimation in Admixed Populations (REAP) software. [27] We considered a related pair if their coefficient was ≥ 0.1 (first- and second-degree relatives). Based on this cut-off, we identified 885 participants as related. Since excluding them would lead to loss of power and possible selection bias, we kept related individuals in the sample and used robust variance estimators to correct results of all multivariate analysis for family structure.

Principal component analysis [28] was used to create a physical health score that included the following measures: systolic blood pressure, body mass index, ratio of total cholesterol to high density lipoprotein cholesterol, hemoglobin value, white blood cell count, log-transformed blood glucose value, log-transformed plasma BNP value (all continuous variables), and *T*. *cruzi* infection (yes or no). Scores may range from - ∞ to + ∞. Higher scores indicated worse health.

We used negative binomial regression (for dichotomous variables) and ordinary least squares regression (for continuous variables) to examine the age and sex (plus family structure) adjusted association between genomic ancestry and ethnoracial self-classification with schooling, household income, health behaviors, physical functioning (all dichotomous variables) and the physical health score (a continuous variable) defined above.

We used mixed-effects logistic regression to estimate odds ratios and 95% confidence intervals to model baseline and 10-year SRH trajectory, using poor SRH as the reference category. [29] To take into account dropouts that could be related to the outcome, we applied the pattern mixture model. [30] This entailed creating a factor variable representing the time of the last valid observation and including it in statistical models as both a main effect and an interaction term with the time variable. We examined the significance of the effect of multiplicative interactions between ancestry in tertiles and self-classification on SRH trajectories, by means of cross-product terms with time. Models were adjusted for the sociodemographic and health factors identified above.

We estimated adjusted hazard ratios and 95% confidence intervals for the association between baseline SRH and time dependent SRH, and subsequent mortality, using Cox proportional hazards models [31] after confirming that the assumption of proportionality among the hazards was met; time scale was number of years since the baseline We examined separately the influence of each genome-wide ancestry and ethnoracial self-classification on this association. All hazard ratios were adjusted for age, sex, schooling, household income, health behaviors, physical functioning and physical health score, as previously described. Additionally, we examined the significance of the effect of multiplicative interactions between each genomic ancestry tertile and baseline and SRH trajectories on subsequent mortality by means of cross-product terms in fully adjusted Cox proportional hazards regression models. We used a clustered robust variance estimate in these analyses to take into account clustering of individuals within families. Finally, we conducted supplementary analyses, based on R squared estimates, to quantify how much of the variation in the outcome (survival time) is explained by baseline SRH and SRH trajectory, respectively [32].

The multivariable models (described above) were build separately for each tertile of genomic ancestry and for ethnoracial self-classification. Separate analyses were performed because African, Native American and European ancestry proportions are complementary measures, whose sum is equal to 1. Further, ethnoracial self-classification showed a strong correlation with each of the genomic ancestries in exploratory analysis. Thus, we opted for not controlling self-classification for ancestry and vice-versa.

Statistical analyses were conducted using Stata 13.1 statistical software. All p-values were 2-tailed (alpha = 0.05).

## Results

### Descriptive

**Fig 1:**
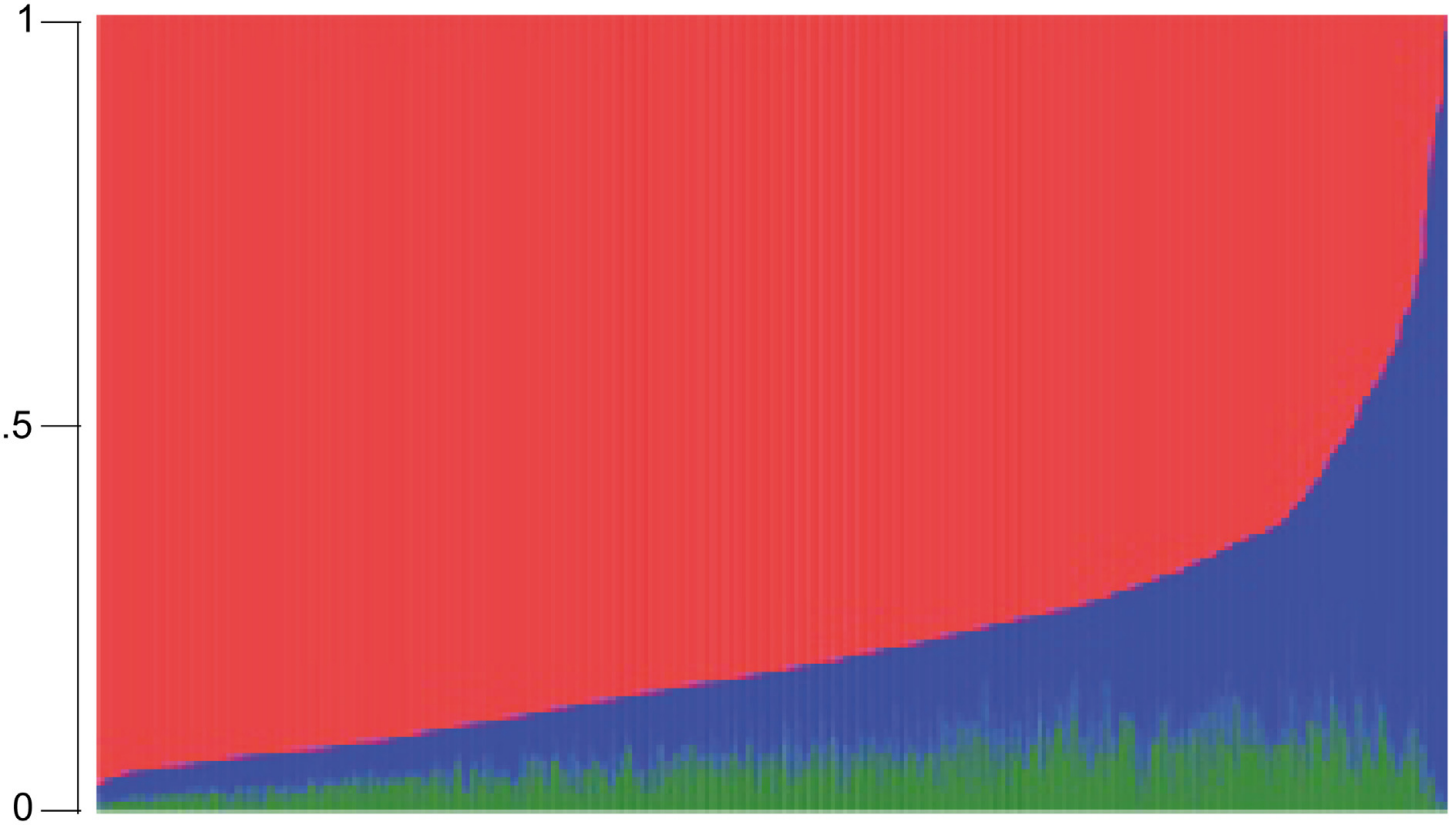
Tri-Hybrid Genome-Wide Individual Proportion of Ancestry (n = 1,311), Bambui Cohort Study of Ageing. The red, blue and green colors represent the European, African and Native American ancestry proportions, respectively.

Of the 1,606 baseline cohort participants, complete data on all study variables were available for 1,311 persons, who were included in the current analysis. As can be seen in Table 1, the mean age of participants at baseline was 68.1 years and 61.4% were women. European ancestry predominated, followed by African and Native American (median = 84.0%, 9.6% and 5.3%, respectively), and prevalence of self-identified Non-White (Mixed or Black) was 39.8%. Most participants had some degree of admixture of African, European and Native American ancestry, as shown in Fig 1.

**Table 1:** Baseline Characteristics of Study Participants, (n = 1311), The Bambui Cohort Study of Aging.

Characteristics	Mean (SD) or Percentage or Median (IQR)
**Proportion of genome-wide ancestry and ethnoracial self-classification**	
African, median (IQR)	9.6 (4.8, 17.5)
European, median (IQR)	84.0 (74.1, 91.4)
Native American, median (IQR)	5.3 (2.7, 8.3)
Non-White self-classification ^a^, %	39.8
**Sociodemographic and health indicators**	
Age in years, mean (SD)	68.8 (6.9)
Female sex, %	61.4
<4 years of schooling, %	63.1
Monthly household income per capita < USD 180.00, %	47.0
Current smoking, %	17.3
Physical exercise <3 times per week, %	86.3
Common mental disorders ^b^, %	37.7
Disability in activities of daily living ^c^	9.8
Systolic blood pressure in mmHg, mean (SD)	137.5 (22.2)
Body mass index in kg/m^2^, mean (SD)	25.2 (4.9)
Total cholesterol/high density lipoprotein cholesterol ratio	5.1 (1.8)
Blood glucose in mg/DL, median (IQR)	99 (91–112)
B-type natriuretic peptide in pg/mL, median (IQR)	79 (42–148)
Hemoglobin in g, %	14.5 (1.4)
White blood cell count, cells/mL x 1,000, mean (SD)	6.8 (2.2)
*Trypanosoma cruzi* infection, %	36.7

Table abbreviations: IQR, interquartile range; SD, standard deviation.

^a^ Black or Brown/Mixed (“Pardo” in Portuguese)

^b^ General Health Questionnaire score ≥ 5

^c^ Feeding, dressing, bathing or showering, toileting, getting in and out of the bed and/or walking across a room

Overall, 18.5% of participants rated their health as poor or very poor. As shown in Table 2, there was a significant increase (p<0.001 in the chi-squared test for trend) in the prevalence of poor SRH ranging from 13.3% among people at the lowest levels of African ancestry to 19.5% among those at intermediate levels to 19.5% among those at the highest tertile of African ancestry (28.9%); a similar trend (p<0.001) was found for tertiles of Native American ancestry (12.8%, 18.8%, 24.0%; p<0.001). In contrast, persons at the highest tertile of European ancestry were significantly (p<0.001) less likely to report poor health (13.0%) relative to those at the intermediate (19.5%) and lowest tertiles (23.1%). Self-classified Non-White persons were significantly (p<0.05) more likely to report poor health (21.8%) compared to those who self-classified as White (16.4%).

**Table 2:** Sociodemographic and Baseline Health Characteristics by Genome-Wide Individual Proportion of African, European and Native American Ancestry and Ethnoracial Self-Classification (n = 1,311), Bambui Cohort Study of Ageing.

Ancestry in tertiles ^a^ or ethnoracial self classification	Age-sex adjusted Coefficient (95% CI)						
	<4 years of schooling vs. higher ^b^	Household income per capita < USD 180.00 vs. higher ^b^	Current smoking vs. no ^b^ ^,^ ^c^	Physical exercise <3 vs. ≥3 times per week ^b^ ^,^ ^c^	Common mental disorders vs no ^b^ ^,^ ^c^	ADL disability vs no ^b^ ^,^ ^c^	Objective health score ^d^ ^,^ ^e^
**African ancestry**							
Intermediate vs. lowest	0.81 **	0.31 *	0.07	0.29	0.16	-0.00	-0.20 *
	(0.48, 1.13)	(0.02, 0.60)	(-0.28, 0.43)	(-0.10, 0.68)	(-0.14, 0.45)	(-0.49, 0.48)	(-.0.37, -0.03)
Highest vs. lowest	1.29 **	0.67 **	0.11	0.32	0.23	0.10	-0.45 **
	(0.91, 1.66)	(0.38, 0.97)	(-0.25, 0.48)	(-0.07, 0.71)	(-0.05, 0.52)	(-0.35, 0.55)	(-0.62, -0.27)
**Native American ancestry**							
Intermediate vs. lowest	0.69 **	0.41 *	-0.10	0.35	0.19	0.12	-0.02
	(0.38, 0.99)	(0.10, 0.72)	(-0.50, 0.29)	(-0.01, 0.71)	(-0.10, 0.47)	(-0.33, 0.57)	(-0.15, 0.19)
Highest vs. lowest	1.84 **	0.71 **	0.33	0.76 **	0.41 **	0.10	-0.26 **
	(1.49, 2.19)	(0.41, 1.00)	(-0.05, 0.70)	(0.36, 0.17)	(0.13, 0.70)	(-0.36, 0.55)	(-0.44, -0.09)
**European ancestry**							
Intermediate vs. lowest	-0.57 **	-0.36 *	-0.14	-0.15	-0.14	0.01	0.31 **
	(-0.94, -0.20)	(-0.65, -0.07)	(-0.52, 0.24)	(-0.53, 0.24)	(-0.42, 0.15)	(-0.44, 0.46)	(0.13, 0.48)
Highest vs. lowest	-1.59 **	-0.65 **	-0.39 *	-0.46 *	-0.33 *	-0.19	0.44 **
	(-1.96, -1.22)	(-0.94, -0.35)	(-0.77, -0.02)	(-0.85, -0.06)	(-0.61, -0.05)	(-0.67, 0.28)	(0.26, 0.62)
Ethnoracial self classification (Non-White vs. White)	0.55 ***	0.11	0.48 **	0.20	0.37 **	-0.27	-0.18 *
	(0.30, 0.80)	(-0.12, 0.34)	(0.16, 0.79)	(-0.14, 0.54)	(0.14, 0.61)	(-0.65, 0.11)	(-0.32, -0.04)

^a^ there were 437 individuals in each tertile of African, European and Native American ancestry group, respectively.

^b^ estimated by negative binomial regression;

^c^ as defined in Table 1;

^d^ estimated by linear regression;

^e^ systolic blood pressure, body mass index, ratio of total cholesterol to high density lipoprotein cholesterol, hemoglobin value, white blood cell count, blood glucose, plasma BNP value and *T*. *cruzi* infection.

* p<0.05;

** p<0.01;

*** <p<0.001

### Age-sex adjusted analysis of the association between genomic ancestry and ethnoracial classification with socioeconomic and health indicators

As shown in Table 2, European ancestry was significantly associated with all sociodemographic and health measures considered in the current analysis (except physical functioning), with better conditions among those at the highest tertile. Both African and Native American ancestry were significantly associated with lower schooling, lower income and worse physical health. Highest level of Native American ancestry (but not of African ancestry) was associated with less physical exercise and higher prevalence of common mental disorders. Those self-classified as Non-White, relative to Whites, were more likely to have lower schooling level, to be current smokers, to report common mental symptoms and to have worse physical health.

### Multivariable analysis of the association genomic ancestry and ethnoracial classification with self-rated health

Over the 10-year follow-up, 9,721 measures of SRH were made (median = 7.4 per participant). Table 3 presents results from multivariate mixed regression models explaining baseline and changes in SRH over time. The results show that the association between poor SRH at the baseline and each genomic ancestry group remains largely significant after adjustments for schooling, education, lifestyle, mental health, physical functioning and physical health score, besides age and sex. Those in the highest tertile of African ancestry were about twice as likely to report poor health (Odds ratio [OR] = 1.98; 95% CI 1.34, 2.93) compared with those in the lowest tertile of African ancestry. For Native American ancestry, both intermediate and highest ancestry levels were significant and suggested a graded relationship (OR = 1.71; 95% CI 1.13, 2.60 and OR = 2.28; 95% CI 1.54, 3.38, respectively). European ancestry showed the inverse relationship with negative and statistically significant associations with poor SRH among those at the intermediate and highest tertile levels (OR = 0.68; 95% CI 0.47, 0.99 and OR = 0.51; 95% CI 0.35, 0.74). The corresponding analysis for ethnoracial self-classification revealed a significant association between Non-White classification and the likelihood of poor SRH at baseline (OR = 1.47; 95% CI 1.08, 1.99).

**Table 3:** Results of the Multivariable Analysis of the Association Between Baseline and 10-year Trajectory of Self-Rated Health (SRH) With Genome-Wide Individual Proportion of African, European and Native American Ancestry and Ehnoracial Self-Classification (n = 1,311), Bambui Cohort Study of Ageing.

Ancestry in tertiles ^a^ or ethnoracial self classification	Mixed-Model Odds Ratio Estimate (95% CI) ^b^		
	Time ^c^	Baseline SRH(Poor vs. Better)	10 Year of SRH Trajectory(Poor vs. Better) ^d^
**African ancestry**			
Lowest	1.08 (0.82, 1.42)	1.0	1.0
Intermediate	-	1.30 (0.85, 1.98)	0.98 (0.91, 1.06)
Highest	-	1.98 (1.34, 2.93) *	0.92 (0.86, 0.99) *
**Native American ancestry**			
Lowest	1.10 (0.84, 1.45)	1.0	1.0
Intermediate	-	1.71 (1.13, 2.60) *	0.94 (0.87, 1.02)
Highest	-	2.28 (1.54, 3.38) **	0.94 (0.87, 1.01)
**European ancestry**			
Lowest	1.02 (0.78, 1.33)	1.0	1.0
Intermediate	-	0.68 (0.47, 0.99) *	1.01 (0.94, 1.10)
Highest	-	0.51 (0.35, 0.74) **	1.07 (1.00, 1.15) *
**Ethno-racial self-classification**			
White	1.04 (0.80, 1.36)	1.0	1.0
Non-White	-	1.47 (1.08, 1.99)	0.99 (0.93, 1.05)

^a^ there were 437 individuals in each tertile of African, European and Native American ancestry group, respectively;

^b^ Adjusted for age, sex, schooling, household income per capita, current smoking, physical exercises, common mental disorders and activity of daily living disability (all dichotomous variables) and health score (a continuous variable) as defined in Table 2. Time scale was number of years since baseline.

^c^ Reference for each ancestry group;

^d^ interaction between ancestry tertile and time on SRH trajectory

* p<0.05;

** p<0.01

With regards to SRH trajectories, Table 4 shows a significant but modest improvement in SRH trajectories among those at the highest tertile of African ancestry relative to those in the lowest tertile (OR = 0.92; 95% CI 0.86, 0.99), and a modest worsening in SRH among those at the highest European ancestry level (OR = 1.07; 95% CI 1.00, 1.15). No significant association was found for Native American ancestry or ethno-racial self-classification.

**Table 4:** Results of the multivariable analysis of the association between baseline and self-rated health trajectory with 10-year mortality, stratified by wide-genome African, Native American and European ancestry (n = 1,311), Bambui Cohort Study of Ageing.

Ancestry in tertiles ^a^	Baseline Self-Rated Health				Hazard ratio (95%CI) ^b^	
	Poor		Good/Fair			
	No. Deaths	Deaths rate per 1,000 pyrs	No. Death	Deaths rate per 1,000 pyrs	Poor baseline Self-Rated Health	Trajectory of Self-Rated Health
**African ancestry**						
Lowest	27	52.8	153	44.1	1.19 (0.77, 1.85)	3.03 (2.16, 4.24) ***
Intermediate	49	71.4	154	42.0	1.55 (1.09, 2.20) *	3.08 (2.14, 4.45) ***
Highest	54	68.9	135	42.9	1.49 (1.06, 2.10) *	3.61 (2.56, 5.09) ***
**Native American ancestry**						
Lowest	28	57.9	153	43.7	1.17 (0.76, 1.81)	3.37 (2.39, 4.76) ***
Intermediate	50	76.6	147	46.1	1.56 (1.12, 2.18) **	3.29 (2.35, 4.60) ***
Highest	52	61.5	122	39.9	1.53 (1.08, 2.17) *	3.15 (2.17, 4.58) ***
**European ancestry**						
Lowest	57	72.9	134	43.7	1.62 (1.17, 2.25) **	3.65 (2.57, 5.17) ***
Intermediate	44	62.8	131	41.0	1.32 (0.90, 1.92)	2.99 (2.05, 4.35) ***
Highest	29	58.8	157	45.1	1.26 (0.84, 1.89)	3.17 (2.29, 4.38) ***
**Ethnoracial self classification**						
White	70	66.0	267	44.6	1.47 (1.10, 1.97) *	3.18 (2.45, 4.14) ***
Non-White	60	65.1	155	41.2	1.37 (0.99, 1.90)	3.44 (2.50, 4.73) ***

Abbreviations: CI, confidence interval; Pyrs: Person-Years at Risk

^a^ there were 437 individuals in each tertile of African, European and Native American ancestry group, respectively;

^b^ Hazard ratios and 95% confidence intervals were estimated by Cox regression and adjusted for age, sex and baseline schooling, household income per capita, current smoking, physical exercises, common mental disorders and activity of daily living disability (all dichotomous variables) and health score (a continuous variable) as defined in Table 2. Time scale was number of years since the baseline.

* p<0.05;

** p<0.01;

*** p<0.001

### Multivariable analysis of the association between self-rated health and mortality

During a mean follow-up period of 8.9 years, 522 participants died and 84 were lost, leading to 11,725 person-years of observation. Overall, the mortality rate was 65.6 per 1,000 person-years among persons who reported poor SRH at baseline compared to 43.3 per 1,000 among those who reported better health. As shown in Table 4, in Cox proportional hazard models that were adjusted for all sociodemographic and health characteristics, participants who reported poor health at baseline were at significantly increased risk for subsequent mortality relative to those who reported good/fair health in the following groups: individuals who were at the intermediate African ancestry (hazard ratio [HR] = 1.55; 95% CI 1.09, 2.20) and the highest tertiles of African ancestry (HR = 1.06; 95% CI 1.06, 2.10); individuals who were at intermediate (HR = 1.56; 95% CI 1.12, 2.18) and highest tertiles of Native American ancestry (HR = 1.56; 95% CI 1.08, 2.17); among those who were at the lowest tertile of European ancestry (HR = 1.62; 95% CI 1.17, 2.25), and for self-classified as White (HR = 1.47; 95% CI 1.10, 1.97). With regards to the prognostic value of SRH trajectories for subsequent mortality, significant and highly similar hazard ratios were found for all groups with values ranging from 2.99 (95% CI 2.05, 4.35) to 3.65 (95% CI 2.57, 5.17). Notably, there was no significant interaction (p>0.05) between genomic ancestry or ethnoracial classification on the predictive value of both baseline and SRH trajectories for subsequent mortality.

Overall, the variation in mortality (survival time) explained by repeated measures of SRH was about 40% higher (R^2^ = 0.41; 95% CI 0.35, 0.49) compared with a single measure of SRH at the baseline (R^2^ = 0.29; 95% CI = 0.24, 0.36). The corresponding values ranged from 0.38 (95% CI 0.28, 0.53) to 0.45 (95%CI 0.36, 0.59) across genome-wide ancestry and ethno-racial self identified groups for SRH trajectory, and from 0.25 (95% CI 0.17, 0.40) to 0.34 (95% CI 0.26, 0.49) for a single baseline measure of SRH.

## Discussion

Major results of this study demonstrate that: first, persons at higher levels of African and Native American genomic ancestry, as well as those self-identified as Non-White, were more likely to report poor health at baseline than other groups, even after controlling for socioeconomic conditions, health behaviors, mental health, physical functioning and a wide array of objective measures of physical health; second, given that baseline SRH was the strongest predictor of health assessment trajectories, those with higher African and Native American genomic ancestry, and those self-identified as “Non-White” tended to report worse health over time relative to their respective reference groups; third, neither genomic ancestry nor ethnoracial self-identification modified the strong predictive power of SRH trajectories for 10-year mortality risk.

The Bambui population is ethnically mixed, linked to a history of decimation of Native American groups (or its incorporation by miscegenation), the importation of slaves from Africa, and colonization by Europeans and their descendants. [33] Our results show that the current genetic make up of the Bambui cohort members reflects this background. Interestingly, the individual proportion of genomic African ancestry among cohort participants (9.6%), and the proportion of European ancestry (84.0%) were similar to that estimated for the Brazilian general population (9.0% and 82.0%, respectively). [34] The contribution of Native American ancestry to the cohort population was smaller (5.3%) than that estimated for Brazil (9%) since our sample does not include the Amazon region, where the largest concentration of people with Native American ancestry resides. [34]

Previous research has shown that adult Brazilians who self-report as Black and/or Mixed are more likely to report worse health outcomes. [8, 10, 35] Our results are in line with these reports and add to them by showing that; 1) ethnoracial disparities in health may occur even in small, tightly-knit populations with relatively small differences in education and income levels; and 2) ethnoracial disparities were apparent not only in schooling and income, health behaviors and mental health-factors that can be observed by individuals themselves and by other people around them, but also in laboratory and other objective health measures.

A major explanation for socioeconomic disparities associated with African ancestry in Brazil is the cumulative effect of the lack of social policies designed to support the newly freed slaves and their descendants since the abolition of slavery in 1888 up to the last decades of the 20^th^ century. [11,36] As a consequence, persons of African origin are more likely to have lower income and education [8, 11]. They also experience race-based discrimination in other realms of life. [37, 38] The most comprehensive studies in Latin American investigating the association between SRH and ethnoracial classification concluded that socio economic differences fully mediate this association. [8, 9] Our results show a different picture. In the current analysis, based on older adults with low socioeconomic and educational levels, poor SRH remained associated with higher levels of African and Native American ancestries and non-White self-identification, independent of education, household income and several health indicators.

As a consequence of sustained marginalization, indigenous populations through Latin America tend to live in more isolated communities and experience higher rates of mortality and morbidity than the general population. [37, 38] Individuals with indigenous parentage may sometimes prefer to self-identify as White or Mixed (“*pardo*”in Portuguese or *mestizo* in Spanish), especially if they live in urban areas. [9, 38] Therefore, self-reports tend to underestimate Native American ancestry. This is true for the Brazilian population as a whole (only 1% of the total population declared being indigenous in the most recent census [12]), as well as for the Bambui cohort population, in which no participant identified himself as being Indigenous. Previous studies showed poorer SRH in persons self-identified as indigenous and/or having indigenous parentage. [9, 38] Our results showed a graded increase in the likelihood of poor SRH among people with increasing levels of Native American ancestry, independent of other relevant factors.

Self-rated health appears to be a universal predictor of mortality. This association has been reported in very different populations and countries [1–3], including Brazil. [4,17] Why SRH has prognostic value for mortality is not well understood [2]. The most intuitive explanation is that SRH reflects an individual’s awareness of symptoms, diagnoses, or diminished functioning, all of which are associated with mortality risk. [2] Others argue that SRH represents a broader dimension of health than do these domains, and its ability to predict mortality is because SRH reflects the general state of the human organism [2]. There are within group differences in the ability of SRH to predict mortality. SRH seems to be a stronger predictor for Whites than for other groups, at least in the United States. [39] SRH appears to be a weaker predictor of mortality in older compared to younger age groups, possibly because of new and/or life-threatening health events with advancing age [2, 40, 41]. To our knowledge, no previous study has investigated the influence of genomic ancestry, as well as that of ethnoracial self-classification, on the ability of SRH to predict mortality in Latin America. To approach the subject we used to two measures of SRH, i.e. a single measure of SRH at baseline and SRH trajectory a measure that captures changes over time. Our results indicate that SRH trajectory is a stronger predictor of mortality for all genomic ancestry and ethnoracial categories than the traditional SRH single baseline measure. Increased risks for mortality associated with worse SRH trajectory were strong and remarkably similar (hazard ratio ~3) across all genomic ancestry and ethnoracial groups.

This study has strengths and limitations. Strengths of the study include the community-based sample, standardized and systematic measurement of parameters at baseline, annual measures of SRH, continuous surveillance of mortality according to standardized criteria, and minimal loss of participants to follow-up. Another major strength is the use of genome-wide measures of ancestry. Genomic ancestry does not change over time, while self-classification is prone to misclassification, particularly in admixed populations, and can even fluctuate over time, due to changing social norms. [11] Previous Latin American studies were cross sectional in nature and considered few health conditions as potential confounders (all of them self-reported) for the association between ethnoracial classification and SRH. [8–10] Our study incorporated a much more robust set of measures of determinants of SRH, allowing us to make more meaningful estimates of ancestry/ethnoracial classification and its association with SRH and mortality over time. However, even given the range of health and health-related variables included in this study, there may be additional unmeasured factors that confound our results. Thus, we cannot discard the possibility of the existence of other variables affecting our results, including unknown genetic factors that might predispose an individual towards poor SRH.

In conclusion, our analyses provide new insights based on solid data on the association between genomic ancestry/ethnoracial and SRH, as well as on its prognostic value for mortality. Our results demonstrate for the first time that higher levels of African and Native American genomic ancestry–and the inverse for European ancestry–were strongly correlated with worse SRH in a Latin American admixed population. Further, genomic ancestry/ethnoracial classification did not modify the strong predictive power of SRH for mortality. Further research is needed to identify the set of mechanisms by which ethnoracial ancestry and the social constructions of ethnoracial ethnic identities may interact to cause the observed disparities in SRH.
